# A Qualitative Study of Indian Women’s Experiences with Sexual Pleasure and Masturbation

**DOI:** 10.1007/s10508-026-03436-y

**Published:** 2026-05-27

**Authors:** Shahzarin Khan, Ngawang Tenzin, Supriya Ingle, Lucia Guerra-Reyes, Satyajeet Sarfare, Siya Kulkarni-Schaefer, Debby Herbenick

**Affiliations:** 1 Department of Applied Health Science, School of Public Health-Bloomington, Indiana University, 1025 E. Seventh Street, Suite 111, Bloomington, IN 47405 USA; 2 The Center for Sexual Health Promotion, School of Public Health-Bloomington, Indiana University, Bloomington, IN USA; 3 Luddy School of Informatics, Computing, and Engineering, Indiana University, Bloomington, IN USA; 4 School of Education, Indiana University, Bloomington, IN USA

**Keywords:** Masturbation, Pleasure, Sex education, COVID-19, Sexual health, India

## Abstract

Masturbation is associated with positive sexual development. However, there is limited research on masturbation in India, where sexual health efforts center on disease prevention and often neglect sexual pleasure. In this study, we aimed to examine Indian women’s experiences of masturbation and sexual pleasure during the COVID-19 pandemic. In 2021, we conducted 22 semi-structured virtual interviews with women aged 20–43, based in India. Interviews were conducted in English, Hindi, and Marathi and were transcribed and translated into English, as needed. Thematic analysis was employed with an inductive coding approach (Nowell et al., 2017). We discerned three themes: (1) ways of learning about masturbation and associated emotions, (2) cultural regulation of women’s sexuality, and (3) change in sexual life due to the COVID-19 pandemic. We found that participants faced early barriers to masturbation due to limited access to sexual knowledge and the absence of comprehensive sex education. Families and social norms stigmatized women’s sexual pleasure, limiting their agency and discouraging self-exploration. The COVID-19 pandemic intensified these barriers by reducing privacy and increasing surveillance, yet participants found ways to explore self-pleasure and masturbation. Our findings challenge the Indian cultural silence and stigma around women’s masturbation by showing that it is not just a private act, but one that is shaped by sociocultural norms and used by participants to reclaim their sexual agency. Our findings highlight the need for sexuality education and public health strategies that are culturally responsive, comprehensive, and pleasure centered.

## Introduction

Much of the extant research on solitary masturbation has focused on addressing empirical questions related to masturbation prevalence and frequency, reasons for masturbation, shame or guilt associated with masturbation, as well as how solitary masturbation is situated within the context of people’s romantic and/or sexual relationships (Bowman, 2014; Gerressu et al., 2008; Herbenick et al., 2023). Many studies on masturbation focus on predominantly White, upper-income heterosexual populations (Bohmer et al., 2022). As a result, little is known about masturbation among other populations (Kaestle & Allen, 2011) with few studies examining diverse populations and international contexts (Chowdhury et al., 2019; Huong & Liamputtong, 2018; Meiller & Hargons, 2019; Thorpe et al., 2023; Wang et al., 2007). Recent studies from Africa, Malaysia, and Brazil critique the reliance on quantitative measures and medicalized framings of masturbation, which often conceals women’s lived experiences (Fiaveh, 2024; Phuah et al., 2024; Soares et al., 2024). These scholars call for gender-equitable, culturally grounded, and pleasure-affirming research that go beyond behavioral indicators such as prevalence and frequency. They emphasize that emotions associated with masturbation, including shame, guilt, curiosity, and empowerment, are shaped by cultural norms, family expectations, religious beliefs, and gendered roles. Hence, advancing an inclusive understanding of sexual pleasure requires examining masturbation as an emotional and social practice.

Masturbation has been associated with more positive mental and physical health (e.g., improved sleep, stress reduction, increased self-esteem, more positive body image) as well as sexual health (e.g., sexual functioning, sexual satisfaction) (Coleman, 2003). Despite these positive aspects, masturbation is often stigmatized and considered taboo in various countries, including in India. Historically, the focus of sexual and reproductive health research in India has been on population control and disease prevention, leaving little known about Indian women’s experiences with sexual pleasure or masturbation (Edmunds & Gupta, 2016; Sawant et al., 2024; Unnithan, 2022). Moreover, patriarchal and sociocultural norms exert control over girls’ bodies from an early age, framing sexual passivity as moral expectations (Sur, 2021). These norms discourage open conversations about sex, promote abstinence until marriage, and suppress women’s sexual desire, resulting in shame and guilt (Dodia, 2023; Edmunds & Gupta, 2016; Narayanan, 2015). In contrast to men, women face gendered double standards that limit their sexual agency and access to pleasure (Krishnan, 2018; Kumari & Siotra, 2023; Singh et al., 2020). Addressing this gap in sexuality research requires examining how cultural and patriarchal frameworks restrict Indian women’s sexual agency and access to pleasure.

The limited available research on Indian women’s masturbation reveals low awareness about masturbation as a healthy sexual practice, stigma around masturbation, and misconceptions shaped by cultural beliefs about female purity and shame. Many women are socialized to believe that masturbation weakens the body, threatens fertility, or undermines future marital harmony (Abraham, 2001; Sachdev, 1998; Sharma & Sharma, 1998). These misconceptions contribute to emotional responses such as shame, fear, and guilt, among those who report masturbating.

At least in convenience samples, about 30 to 60 percent of Indian women have engaged in masturbation; however, many of these women hold negative views and misconceptions about the practice (Abraham, 2001; Kar & Koola, 2007; Sachdev, 1998; Sharma & Sharma, 1998). In one of the earliest studies of masturbation among Indian women, Sharma and Sharma (1998) found in a sample of 530 Indian unmarried heterosexual college women, that 30% of these women had engaged in masturbation and all had some misconceptions regarding masturbation including beliefs that masturbation caused weakness, disease, infertility, and marital disharmony. These findings align with other studies, such as Sachdev (1998), which found that 52% of Indian women in their sample engaged in masturbation, though only 38% thought masturbation was a healthy practice. Also, Kar and Koola (2007), in a study of 61 participants in a small Indian town, found that about 60% of the women in their sample had ever masturbated, and none of the unmarried women reported having ever experienced orgasm. In another study of young Indian women and men studying in colleges and who were from low-income backgrounds, most women did not show any awareness of masturbation and, when it was explained to them, they expressed surprise and considered such an act as ‘shameful,’ ‘abnormal’ and ‘dirty’ (Abraham, 2001). One qualitative study with Indian women found that cultural expectations of female purity and motherhood framed women’s masturbation as inappropriate, leading to shame (Narayanan, 2015). Lastly, a recent comparison study of 60 masturbating and 60 non-masturbating Indian married women found that cultural stigma, family structures, and lack of privacy negatively influenced women’s masturbation practices, while younger age, higher education, urban and nuclear family settings, were linked to greater likelihood of masturbation (Ramasubramanian & Veerabalajikumar, 2024). However, most of these studies are older, focusing primarily on married women reflecting the stigma around premarital sex and none of the samples include queer or lesbian women. As a result, these studies overlook recent shifts, including expanded internet access (Majumdar, 2023), the use of social media as spaces for discussing sex, and the growing availability of Indian sex toys. Together, these developments contribute to a sex-curious discourse among younger generations (Shetty, 2022; Vaidya, 2021).

Additionally, the COVID-19 pandemic and its resulting limitations on physical contact during lockdown influenced people’s sexual behavior in India as has been shown elsewhere (Alinaghi et al., 2025; Qalati et al., 2022). Globally, the COVID-19 pandemic and related lockdowns decreased partnered sexual activity (Masoudi et al., 2022). Recent studies highlight the heightened stress, restricted physical contact, and increasing interest in solo sexual activities and sex toys in India (Mhatre et al., 2025; Roy et al., 2021). These shifts related to the pandemic highlight the need to explore how India’s gendered and sociocultural norms shaped women’s masturbation experiences and attitudes toward self-pleasure under conditions of social isolation.

Previously mentioned masturbation studies used quantitative surveys that show that while many Indian women report masturbating; they often associate masturbation with guilt. Meanwhile, qualitative studies on Indian women’s sexual pleasure, such as Chertow (2019), Dodia (2023), and Rajan and Pathak (2024) have examined sociocultural norms around desire but remain focused on partnered or marital contexts, leaving masturbation as a solitary activity largely invisible There is a lack of qualitative research examining how Indian women learn about masturbation, the emotions they associate with it, and how the COVID-19 pandemic shaped these experiences. Addressing these gaps is essential to understanding how shifting social norms are transforming sexualities among Indian women. Increased internet access, social media platforms, and digital sex education spaces have expanded opportunities for young women to encounter information about masturbation and sexual pleasure (Majumdar, 2023; Shetty, 2022; Vaidya, 2021). Emerging sex-positive discourse, including conversations about women’s pleasure, sex toys, and consent, has begun to challenge norms that frame women’s sexuality as passive or solely marital. Scholars call for culturally sensitive and sex-positive research on masturbation that integrates psychological, social, and relational dimensions (Neena et al., 2024; Ramasubramanian & Veerabalajikumar, 2024).

Through this exploratory qualitative interview study, we sought to understand Indian women’s experiences with masturbation during the COVID-19 pandemic. Specifically, we examined: How do Indian women learn about masturbation, what emotions do they associate with masturbation, and how have their experiences been shaped by socio-cultural norms and the COVID-19 pandemic?

## Method

### Participants

We followed a qualitative interview study design. Data were collected during the COVID-19 pandemic restrictions in 2021, and thus all interviews were conducted by the third author (SI, who was living in India while conducting interviews) virtually over Zoom. To be eligible for the study, participants had to be (1) between the ages of 18 and 60, (2) currently living in India, (3) identify as women, and (4) have access to the internet.

### Measures and Procedure

The third author (SI) conducted recruitment through social media (Instagram and Facebook) and distributed digital flyers about the study among her networks, with the request for these individuals to share the recruitment messages among their own networks. Interviews were available in English, Hindi, Marathi, and Bangla. Participants received a $25 Amazon gift voucher. We developed a semi-structured interview guide to address the research questions, allowing for the inclusion of additional questions that emerged during the interviews in response to the conversational context. This guide was developed by third author (SI). To ensure validity, the interview questions were reviewed and revised by the two other authors (LG and DH) specializing in women’s sexual health. The guide was organized into four key domains that directly mapped onto the research questions: (1) learning around masturbation, (2) emotional meanings and personal practices, (3) relationships, and (4) the impact of COVID-19–related restrictions. First, questions on learning explored how participants learned about masturbation, the role of peers, partners, migration, and internet use. Second, questions on emotions and individual practices examined participants’ feelings toward masturbation, bodily experiences, use of media or objects, and meanings attached to pleasure and orgasm. Third, relational domains focused on conversations and practices related to masturbation with partners. Finally, COVID-19–specific questions explored how lockdowns, privacy constraints, illness, grief, and economic stress shaped changes in masturbation. The interview guide is attached in Appendix. For this study, we analyzed all the interview domains except relationships. The third author (SI) conducted all 22 interviews between July 2021 and September 2021 online. Considering the nature of the topic on masturbation, we did not get sufficient responses with a younger age group as eligibility criteria and hence we expanded the age range to 18–60 in eligibility criteria to recruit more participants.

All interviews were audio recorded, transcribed and translated into English by the four authors (SK,TN, SS, SK2)who were native fluent in those languages. We distributed the files among the four of us for equal division of labor. Once a file was translated and transcribed by one author, another team member reviewed it for errors. We had regular team meetings to discuss any issues regarding the translation to ensure accuracy. The maximum interview duration was 1 h 28 min, minimum was 34 min and average was 53 min. We arrived at a sample size of 22 based on meaning saturation which was reached when interviews no longer produced new codes, dimensions, or insights (Hennink & Kaiser, 2022; Hennink et al., 2017, 2019; Saunders et al., 2018). After each interview, SI documented analytical memos and developed preliminary codes, assessing whether new aspects or nuances emerged. Saturation was reached when no new interpretive or explanatory information was identified. Additional factors that influenced the sample size included the use of snowball and referral sampling, the homogenous nature of the sample, and practical constraints such as limited funding and time during the COVID-19 pandemic and the sensitive nature of the research topic of women's masturbation.

### Data Analysis

We employed an inductive thematic analysis approach, conducted in six steps (Nowell et al., 2017) and aided by Dedoose. The coding team consists of four team members (SK, TN, SS, SK2). We divided ourselves into two person coding teams. First, we became familiar with the data by engaging in a close reading of interviews. We created initial codes and categories by coding the same initial four interviews and adding emergent codes. Once we had emergent codes, each two-person coding team coded the same 11 interviews resulting in each file being coded twice. The group met every two weeks to discuss emergent codes and harmonize coding structure through consensus. Then, we found themes through in-depth analysis and discussion of the coding results. A subgroup of the coding team met to discuss overall preliminary findings and then analyzed each category to identify general patterns. We discovered higher-order themes using recategorization, diagramming, and close analysis of the quotations generated by code. Next, we reviewed, defined, and named themes through consensus and in consultation with research team members, until six primary themes were identified. Supporting statements for each of the themes were collected from the interviews. Finally, we produced narrative and descriptive results for each of the selected categories of interest. We did not seek statistical inter-rater reliability, given its inappropriateness for thematic analysis, but we employed a consensus-building approach to code and theme development (Braun & Clarke, 2022). We maintained a detailed electronic audit. We tracked the steps taken from data collection, coding, analysis, and interpretation, including versions of the interview guide, lists of initial codes and their meanings, different versions of thematic maps, and detailed notes from team meetings (Carcary, 2009; Lincoln & Guba, 1985).

### Positionality

Our research team, which was comprised of two faculty members and five graduate students, represents a broad multigenerational and international perspective, spanning India, Latin America, and the USA. The faculty members have decades of experience in research on sexual health and qualitative methodologies. Six of our team members are cisgender women, and one is a cisgender male. Team members have prior experience in medical care, nursing, advocacy work, and sexual health research. The four team members who collected and analyzed the data were of Indian origin with higher education, native fluent in Hindi, and socially and culturally from a higher-income stratum of Indian society. This allowed data collection to happen in the language of choice for participants and facilitated cultural closeness. The third author (SI) who did the interviews, shared some intersecting identities with participants, which positioned her as an insider (being an Indian woman) allowing her to build rapport, understand cultural nuances, and conduct interviews in multiple SA languages. At the same time, she occupied an outsider role as a researcher, which shaped her fluency in discussing topics on masturbation that many participants had not explored publicly or even privately before. As a group, the shared positionality of the research team is an interest in addressing sexual health issues that limit women’s ability to lead healthy, fulfilling sexual lives.

## Results

In response to our study aims to examine masturbation experiences among Indian women during COVID-19, we found three major themes: (1) “navigating emotions while learning about masturbation” describes how participants learned about masturbation through informal sources, and how they navigated emotions from early guilt to self-acceptance, (2) “cultural regulation of women’s sexuality” describes sociocultural and familial norms that restrict their sexual self-exploration, and (3) “changes in sexual life due to COVID-19 pandemic” describes the disruption in participants sexual lives, including decreased privacy leading to reduced sexual activity, emotional distress leading to fluctuation of sexual desire and new ways of exploring pleasure. Table 1 summarizes the themes, sub-themes, and their meanings. We found that participants faced barriers to masturbation due to limited access to sexual knowledge in the absence of comprehensive sex education. Stigma around women’s sexual pleasure, reinforced by family, religion, and social norms, restricted participants’ sexual agency and discouraged self-exploration. The COVID-19 pandemic intensified the social constraints by reducing privacy and increasing surveillance, yet participants found ways to explore self-pleasure.

**Table 1:** Themes, sub-themes, and meanings

Theme/sub-theme	Meaning
1. Knowing Masturbation, Navigating Emotions	Participants described how they learned about masturbation and the emotions they attached to it
1.1 Ways of Learning about Women’s Sexual Pleasure	Participants accessed knowledge about masturbation in informal and unequal ways, shaped by class, geography, and digital access, often marked by stigma and silence
1.2 From Shame to Acceptance: Changing Emotions around Masturbation	Participants initially experienced guilt and shame around masturbation, which gradually shifted to acceptance over time
2. Cultural Regulation of Women’s Sexuality	Participants described navigating sociocultural and familial constraints on their sexuality
2.1 Sociocultural Control through Shame	Participants experienced sociocultural norms that framed women’s sexuality as shameful and passive, enforcing moral surveillance and reinforcing gendered double standards
2.2 Family as Gatekeeper through Privacy Constraints and Silence	Participants described how families regulated sexual knowledge and behavior through silence, surveillance, unequal treatment, and denial of privacy, limiting their masturbation experiences
3. Changes in Sexual Life due to the COVID-19 Pandemic	Participants explained how the pandemic disrupted sexual routines and privacy, reshaped sexual desire, and created opportunities for sexual exploration
3.1 Privacy Constraints and Reduced Activity	Participants described how COVID-19 lockdowns and returning to family homes reduced their privacy and limited sexual activity
3.2 Emotional Distress and Changing Desire	Participants reported decreased sexual desire due to emotional distress from the pandemic, with fluctuations based on emotional and environmental stability
3.3 Re-inventing Ways for Pleasure	Participants explored masturbation, digital sex, and online dating as adaptive strategies to reclaim agency and redefine pleasure under constrained circumstances

### Demographics

Twenty-two Indian women participated in the study, all of whom reported being cisgender women. Of these 22 women, 13 described their sexual orientation as heterosexual, four as bicurious, and one as pansexual, among other identities. Participants’ ages ranged from 20 to 43 (mean = 28.6 years). Their age at first consensual partnered sexual experience ranged from 17 to 28 (mean = 21.4). Participants described their occupations as students (*n* = 5), researchers (*n* = 5), doctors (*n* = 3), artists (*n* = 3), and teachers (*n* = 2). All participants were unmarried, with 11 describing themselves as single and 11 as committed to a partner. Participants could describe any kind of solo and/or partnered sexual experiences. In the present study, all participants except one described partnered experiences involving male partners. Table 2 summarizes the demographics covering age, sexual orientation, relationship status, and growing up in rural or urban areas.

**Table 2:** Participant demographics

Characteristic	Type of characteristic	N	Percent
Sexual orientation	Heterosexual	15	68
	Bicurious	4	18
	Heterosexual/Bicurious	2	9
	Pan sexual	1	5
Age (in years)	20–25	7	32
	26–30	9	41
	31–35	3	14
	35–40	2	9
	40–45	1	5
Grew up in rural or urban India	Rural	11	50
	Urban	11	50
Relationship status	Single	11	50
	In a relationship	11	50

#### Theme 1: Navigating Emotions While Learning About masturbation

This theme explores how participants came to learn about masturbation and how they felt about their masturbation experiences. Participants’ journeys were shaped by two processes: (1) accessing information through informal channels, and (2) navigating emotions such as guilt, shame, confusion, and eventual acceptance. These two processes show how sociocultural norms, cultural silence about sex, and the school’s neglect of sex education shaped both what participants knew and how they felt about women’s self-pleasure. This theme reveals the emotional labor participants undertook to understand their bodies. Their narratives show how knowledge of women’s sexual pleasure is unequally distributed, stigmatized, and slowly reclaimed over time.

##### Subtheme 1: Ways of Learning About Women’s Sexual Pleasure

This sub-theme explores how participants encounter knowledge about women’s sexual pleasure and masturbation through informal channels such as the internet, friends, partners, and more. Participants’ accounts highlight how access to masturbation knowledge is shaped by factors like class, geography, digital access, and privacy norms. Participants experienced pleasure before they had the words to make sense of it, revealing a gap between their bodily experiences and what society teaches about sexuality.

Participants described having first learned about masturbation between the ages of 8 and 30 years (mean age = 17.6 years), and they described multiple sources of having learned about masturbation. Table 3 summarizes the data on their primary sources of information on masturbation. The most common way participants learned about masturbation was through online sources, particularly social media and pornography. For example, Participant 3 (20, heterosexual) described having found “porn” videos that depicted partnered sex and then went on to look for further information on masturbation. Others, such as Participant 1 (25, bicurious), described pornography as a practical source of information on how to enjoy self-pleasure. Some participants didn’t provide specific sources and instead used the general term ‘internet’ as a source. In such cases, participants reported searching the internet for information about masturbation or exploring different websites. Internet searches most often occurred through their own devices (cell phones and laptops). However, access to such content was uneven. Participants from small towns or conservative households, like Participant 10 (25, heterosexual), mentioned restricted privacy and often relied on shared devices or cybercafes, limiting their ability to seek information privately. Participants’ accounts reveal how media served as a primary, though uneven, source for learning about masturbation, with access shaped by privacy, family surveillance, and geography.

**Table 3:** Source of information on masturbation

Source of Information	N	Exemplary Quotations
Internet and Porn	16	Participant 3**:** It was very overwhelming in the beginning. Because I had no one to guide me, it was mostly the internet
Accidental or self-exploration out of curiosity	10	Participant 15: I would press my thighs together to feel pleasurable. But I did not see it as masturbation, as I didn’t know about it
Partner	8	Participant 14: I think I found out with the help of my partner. He was older than me, and he had multiple partners, so he was very experienced
Friends	8	Participant 12: Well, first we started talking about the periods. And then someone just said something about the porn movie and suddenly this topic (masturbation) came up
Comics, Magazines, Books, and Movies	7	Participant 16: At my home, a women’s magazine used to come for my mom in Hindi. Once, I read a story about a woman having sex with her husband, and there was a column, and there used to be a column where a counselor would answer sex-related questions
School	2	Participant 7: I think I was in eighth grade. There were talks in school and I was introduced to porn for the first time then. I did not know what masturbation was
Abuse	1	Participant 6: She (My partner) was just there and is much older than me. When I look back, she was probably molesting me. Because I was 16–17 and I didn’t know much about it

Less than half of the participants reported discovering masturbation through self-exploration. Those who did, often described exploring their bodies out of curiosity and not necessarily being aware of the practice as a form of masturbation. For example, Participant 15 (23, heterosexual) described “thigh squeezing as pleasurable, but I did not see it as masturbation.” This highlights how the experience of pleasure often came before participants had words to explain it. For some, friends and peers were a source of information. Participants reported having heard from friends about masturbation and sex, with discussions focusing on their curiosities, erotic stories, and materials, but not explicitly on masturbation techniques. Some participants had first learned about masturbation from their partners and then continued exploring on their own, often with the help of the internet. Others mentioned books, comics, and magazines as sources of learning about masturbation. For example, Participant 5 (29, bicurious) reported learning about squirting through the women’s magazine “Cosmopolitan,” whereas Participant 12(33, heterosexual) talked about learning from “romantic scenes” in movies. Few participants described having heard about masturbation for the first time through health talks arranged by the school, which was an uncommon source of learning. Lastly, one participant mentioned that she was introduced to masturbation through sexual abuse as a child, an experience distinct from the majority but important in understanding the diverse ways through which participants come to know their bodies. These accounts show that participants learned about masturbation through informal sources, highlighting how sexual knowledge often emerged from viewing media.

Most participants reported having had no formal comprehensive sexuality education at school, and that there had been no discussion about sexual pleasure at school. As Participant 21 (27, heterosexual)mentioned, even if sexuality education was provided in school, “nothing significant gets delivered,” and “it was very theoretical,” and no practical knowledge was provided. Participants reported that most of the content covered in schools was on the topic of periods and reproduction, and was abstinence-based, and even then, sometimes chapters on reproduction were skipped in class, even though it was a compulsory part of the syllabus. Participant 13 (22, heterosexual) talked about the lack of initiative from teachers and educators—“the teachers’ voice would go low.” Participant 10 (25, heterosexual) mentioned, “teachers feel weird to talk about it and are not comfortable teaching you about it.” Participants’ experiences reveal that school-based sex education failed to address sexual pleasure, pushing them toward informal, often misleading sources.

Overall, this sub-theme reveals that learning about masturbation was developed through informal, uneven, and limited sources, shaped by access to technology, cultural silence, and lack of private space. These experiences also reflect how knowledge about sexual pleasure was influenced by class and geography. The lack of comprehensive sex education from schools highlights the role of the education system in contributing to stigma around sexuality, leading to long-term barriers to sexual pleasure.

##### Subtheme 2: From Shame to Acceptance: Changing Emotions

This sub-theme explores the emotions that accompanied participants’ experiences of masturbation, as they navigated adolescence and adulthood. Many described early feelings of guilt, shame, fear, and confusion, which stemmed from religious teachings, parental responses, misinformation, and societal silences around women’s pleasure. As participants grew older, they described viewing masturbation with more acceptance, replacing guilt and fear with understanding and curiosity.

Several participants reported that their schools or religious environments taught them that masturbation was wrong. For example, Participant 19 (29, heterosexual), who had attended a convent school, shared: “I did not understand if what I was doing was a sin or crime,” illustrating how early messages framed masturbation as punishable. Her feelings of guilt eventually decreased with age, but the emotional residue of being taught that “masturbation is a sin” stayed. This reflects how religious teachings can produce long-lasting internal conflict around self-pleasure. Shame was also reinforced through participants’ sexual partners. Participant 7 (26, heterosexual) described how her boyfriend’s disapproval of masturbation had led her to feel guilty about her behavior. Rather than receiving support for exploring her body, she encountered judgment. This reveals how sexual relationships can also act as another source of surveillance and shame.

Parental reactions further shaped fear and discomfort. One participant described her feelings of shame as she recalled being caught by her mother while masturbating as a child and being told it was wrong. This response framed masturbation as something to hide or be punished for, heightening fear and shame. Another participant reported feeling afraid of becoming infected from masturbating and expressed discomfort with vaginal fluids. This reflects how the lack of familiarity with their body and the lack of sex education contributed to fear. These accounts illustrate how a lack of affirming, body-positive information left participants to navigate confusion alone.

Some participants internalized dominant sexual scripts that positioned penile-vaginal intercourse as the only form of pleasure. Participant 15 (23, heterosexual) shared, “I thought something was wrong with me as this (masturbation) was the only way I could get pleasure. I don’t get pleasure out of penetration.” Her reflection highlights how normative ideas of sexual pleasure can undermine solo or non-penetrative pleasure, making women question their desires and bodies. Despite these challenges, several participants described a gradual shift toward acceptance of masturbation due to various factors such as new friends or partner or new environment such as college. Over time, participants began to feel less guilty, viewing masturbation as “natural and normal” Participant 15 (23, heterosexual). Participant 19 (29, heterosexual) described she “used to feel super guilty” due to her school experience. This eventually changed in college, “when I realized that that’s not a bad thing, its natural. That’s when I stopped feeling guilty.” A few expressed excitement at discovering a pleasurable new sensation. Participant 18 (33, heterosexual) shared how having multiple relationships led to trying different techniques, “gradually, one has multiple relationship, one tries different things. Something magnificent is there, that is yet to be explored. I feel, I gained the perspective with lot of experience.” Another participant mentioned how partner can be helpful in this emotional journey.

I felt that I should not do this (masturbation), but I kept doing it. Slowly as I grew to age of 18–19, when I got my first boyfriend, then I realized that that was good. When I helped myself, gave pleasure to myself, it was even better than being with another person. Participant 17 (30, heterosexual/bicurious)

These accounts illustrate that with greater exposure, age, relationships, participants’ emotional responses shifted from shame to self-affirmation. Overall, participants’ narratives reflect how sexual norms and silence about sex shape not just behavior, but also emotional responses to pleasure.

#### Theme 2: Cultural Regulation of Women’s Sexuality

This theme captures how Indian cultural, patriarchal, and family norms regulated participants’ sexual knowledge, behavior, and access to pleasure. The participants’ accounts reveal that women’s sexuality was framed as dangerous and shameful, resulting in fear, misinformation, and self-restriction. These mechanisms of sexual control by society and family are covered by two sub-themes: (1) sociocultural control through shame, and (2) family as gatekeeper through privacy constraints and silence.

##### Subtheme 1: Sociocultural Control through Shame

This sub-theme captures participants' descriptions of how sociocultural norms in India have framed women’s sexuality as passive, immoral, and shameful. Participants described cultural expectations of virginity, obedience, and purity, reinforcing the idea that women’s sexual desire and agency were dangerous, discouraging self-exploration. These narratives reveal how cultural and patriarchal norms operate as surveillance, producing sexual shame and restricting women’s access to sexual autonomy and pleasure.

All participants except one talked about their sexual experiences within male–female relationships. Participants described the cultural expectations for unmarried Indian women to be abstinent before marriage and to be submissive to their future male partner. Participant 5 (29, bicurious) shared about the expectations for women to be “virgin” and “pure” before marriage, highlighting the sexual control by society on unmarried women’s premarital sexual activity. She also described how Indian women who have sex before marriage are considered “dirty.” Another example of control of women’s sexuality, Participant 6 (38, heterosexual) noted, “It’s society’s understanding that females should not ask for it [sex], not be proactive, and a female partner should give herself to a male partner,” pointing to how women were expected to be sexually submissive. Similarly, Participant 15 (23, heterosexual) observed that sexual freedom is reserved for men but condemned in women: “It is fine if a guy does so, because it is a boy thing. But as a girl, you must suppress emotions and desires.” She described her fears in talking about “sex desires openly” would lead others to judge her character. These narratives reflect a sexual double standard where male sexual desire is accepted and expected, while female desire is monitored, silenced, and judged. The expectation for women to be abstinent before marriage and submissive within heterosexual relationships reveals how cultural norms position women as passive recipients rather than active agents of pleasure. These norms constrain participants’ sexual agency and undermine their right to sexual desire and pleasure.

Participants also described how the cultural framing of sex as shameful led to self-restriction. Participant 10 (25, heterosexual) explained, “We (women) can’t touch ourselves, it is wrong,” and emphasized that many women avoid all sexual exploration until marriage due to internalized shame. Such beliefs, reinforced by family and community expectations, positioned sexual pleasure as something women must defer or deny entirely.

A lot of factors influence women to think that they are not even entitled to pleasure because all that is wrong. There are people (women) who still think that they should only have sex after marriage. And they get married around the age of 25. So, until the age of 25, they would not have tried anything. Participant 10 (25, heterosexual)

This account illustrates how cultural beliefs frame women’s self-pleasure as immoral, which leads participants to delay or avoid sexual exploration. Overall, this sub-theme shows how sociocultural norms suppressed participants’ sexual pleasure. Participants internalized these cultural expectations of sexual passivity, leading to guilt, silence, and self-regulation.

##### Subtheme 2: Family as Gatekeeper Through Privacy Constraints and Silence

This sub-theme captures participants’ experiences of gendered control within Indian families, restricting their access to sexual knowledge, privacy, and bodily autonomy in various ways. Participants described growing up in environments marked by surveillance and silence around sex, where moral policing and unequal treatment of girls constrained access to sexual information and exploration. This sub-theme illustrates how familial regulation reinforced a system of sexual silence and restricted participants’ sexual agency and self-pleasure.

Most participants described their families as ‘conservative’ and indicated that their families did not talk about sex. Many participants reported growing up without any discussion on sex with their parents, and if there was any discussion at all, messages often revolved around the dangers of women’s sexual desire and how it is to be reserved for marriage and reproduction. Participant 6 (38, heterosexual) mentioned that her “parents do not like to talk about sex.” This highlights how family silence around sexuality reinforces participants’ early understanding of sex as shameful. This illustrates how a lack of open discussion in the family may lead to confusion, guilt, and isolation, forcing many to rely on informal sources.

Family control included surveillance of women’s behavior. Participants reported being treated more strictly and suffering harsher consequences than their brothers on matters of sexuality. While male siblings enjoyed greater freedom, participants described being subjected to scrutiny, restriction, and moral judgment. Participant 11 (33, bicurious)recalled being shamed and physically assaulted by her brother for having multiple sexual partners, while his similar behavior was accepted. She said, “My brother said ‘apne hi thali me ched hai’ [there is a hole in our plate]. He meant I am such a bad girl. How I am ruining my family’s reputation. He threw a chappal [footwear] on my face.” Participants’ account highlights the emotional abuse and social exclusion women face for having multiple sexual partners, while men are not only excused for the same behavior but also, feel entitled to shame and punish women, revealing a gendered hypocrisy. These unequal dynamics highlight how patriarchal family structures discipline women’s sexual agency, reinforcing women’s subordination through fear and violence. Participant 21 (27, heterosexual) described how her phone was regularly monitored, while that of her brothers was not. She described how her family controlled her privacy as compared to her brother and added, “It was a big deal to roam around with guys.”

Once my mom saw that I talked to a lot of guys on my phone. She said that you ‘loose talk’ with them a lot. Till I was in the 11th or 12th, my phone used to be checked by my parents. But they didn’t check my brother’s phone Participant 21 (27, heterosexual)

While she reported being monitored and judged for interacting with boys, she noted that her brother’s behavior went unchecked, exposing a double standard that privileges male autonomy and polices female behavior. This highlights how the unequal treatment limits girls’ freedom and sends the message that their actions and desires are wrong or untrustworthy, reinforcing patriarchal control. Another example, Participant 1 (25, bicurious) recalled being told by her mother that boys would “misuse girls,” placing the burden of safety solely on women. Participants talked about how women are taught to protect themselves from men and not engage in any kind of sexual or “provocative” behavior to provoke men, as society blames women if anything goes “wrong”, such as sexual violence or unplanned pregnancy. Due to the silence around sex in their families, participants felt the lack of a safe support system at home to discuss sensitive topics such as unplanned pregnancy, STIs, or violence.

Beyond moral control, participants described how physical space, and privacy played a role in sexual control. Many participants reported lacking private rooms, being questioned for spending time alone, and even using bathrooms. Participant 14 (30, heterosexual) talked about the control of her movement by her family with constant questions on “Where are you going?” and “Cannot be out late in the night.” Many mentioned privacy issues while growing up, and that not having a private room had limited their access to personal activities, including masturbation. While some lived in multigenerational family households, others reported having to share rooms with their siblings or parents. Participant 22 (43, heterosexual) noted the constant presence of family members as a barrier to privacy, and others shared that their internet usage was closely monitored. She talked about “lack of privacy” and “people being always around in the house.” A few participants spoke about using bathrooms as private spaces. However, their privacy was not always respected. For example, Participant 11 (33, bicurious) talked about the questioning nature of parents when she was by herself in the bathroom for even “10–15 min.” A couple of participants (Participants 16 and 21) mentioned the lack of privacy in using and browsing the internet, as parents would control their access to the internet. These accounts reflect how restrictions in physical space and privacy limited participants’ personal freedom and their ability to explore their bodies or access information.

Overall, this sub-theme reflects how families, especially parents, controlled privacy, space, and information to monitor participants’ behavior, denying sexual knowledge, punishing agency, and reinforcing shame, which limited their sexual exploration and pleasure.

#### Theme 3: Changes in Sexual Life Due to the COVID-19 Pandemic

This theme captures participants’ experiences of masturbation and sexual pleasure during COVID-19. Participants described how the pandemic disrupted sexual routines and privacy and reshaped sexual desire. Participants also reported accessing new opportunities of pleasure such as phone sex, sexting and more digital forms of sexual communication. Participants’ experiences reflect how global crises intersect with structural conditions such as housing, mental health, and digital access to reshape sexual pleasure. Participants' masturbation experiences during the COVID-19 pandemic are covered by three sub-themes: (1) privacy constraints and reduced activity, (2) emotional distress and changing desire, and (3) re-inventing ways for pleasure.

##### Subtheme 1: Privacy Constraints and Reduced Activity

This sub-theme captures participants’ experiences of decreased privacy during COVID-19 that led to reduced sexual activity. Participants described living with family reduced privacy, which restricted masturbation and partnered sex, while lockdowns and fear of infection limited casual dating. Participants who were in relationships described that the absence of private space strained both physical and emotional connection. Participants described feeling monitored, stressed, and disconnected from their sexual selves. This sub-theme reflects how the return to family living during the pandemic intensified conditions of social constraint.

Participants described sharing living space with their parents and siblings. Participant 2 (23, heterosexual) said she felt “homeless within her own house” as she had no private room. Participants reported that the absence of private space and individual rooms caused a decrease in masturbation frequency. Participant 3 (20, heterosexual) said, “Whenever I had the urge, I had to wait till midnight or 2–3 am to do it (masturbate).” Participant 17 (30, heterosexual and bicurious) said, “So I would take time and plan when and where I could do it (masturbate).” These accounts reflect how the pandemic intensified existing structural and cultural barriers to sexual agency. The return to shared family spaces reduced opportunities for privacy, especially for those who were unmarried, and reinforced constraints on self-pleasure.

Participants reported that those who were dating or in a relationship, and unmarried, couldn’t meet in person, and, when they did, they lacked private space, leading to a decrease in partnered sexual activity. Participants described feeling fear of new partners due to fear of SARS-CoV-2 infection, lack of trust, lockdown restrictions, and social distancing. Participant 18 (33, heterosexual) said she would “rather not have sex than get a COVID infection.” This reflected the lack of trust in new partners’ COVID status or vaccination status. Participants described difficulties with going outside due to lockdown, as well as inviting new people to one’s home due to social distancing. Participant 10 (25, heterosexual) mentioned how it is “difficult to maintain an open relationship’ during the COVID-19 pandemic. Participant 20 (38, heterosexual)mentioned how the “pandemic put an end to casual dating” due to a lack of trust. These accounts reflect how the pandemic didn’t just interrupt sexual activity, but it also revealed how health fears forced people to find private alternatives. Participants’ narratives point to a broader dynamic where public health risks about COVID-19 magnified private anxieties about sexual health, safety, and trust.

Overall, the pandemic disrupted participants’ sexual lives as living with family restricted access to private space, limiting opportunities for masturbation and partnered sexual activity. At the same time, participants described how their fears of infection reduced partnered sex with new or casual partners. These conditions reflect that social isolation forced participants to delay, suppress, or reconfigure their sexual pleasure within new constraints.

##### Subtheme 2: Emotional Distress and Changing Desire

This sub-theme captures the diverse sexual desires of participants as they navigated feelings of anxiety, grief, and depression during the pandemic. Participants described how their grief, trauma, and collective loss reoriented sexual priorities, making sexual pleasure feel irrelevant. This sub-theme shows how participants’ sexual desire depended on emotional and environmental stability, highlighting how mental health and structural crises shape sexual pleasure.

Many participants described how grief and anxiety due to illness or loss within their families made them uninterested in sexual activity. Many participants said they “were not thinking about sexual stuff.” Participant 7 (26, heterosexual) said, “it was so depressing, seeing so many people die around.” Similarly, Participant 13 (22, heterosexual) expressed fear for her hospitalized mother and said, “Sexual things were out of the picture” and Participant 2 (23, heterosexual) discussed her anxiety around the time of her grandmother’s death and beyond, noting “Even today, when the phone rings, I worry if someone is ill or has passed away.” This reflects how collective trauma, and personal loss led many participants to deprioritize sexual desire, as grief caused emotional detachment from their bodies and reshaped their relationship with sex and pleasure.

Participants described how mental health influenced their sex drive. Participant 11 (23, bicurious) reported depressive symptoms that affected her appetite and mood, from hearing news of deaths within her community, while Participant 22 (43, heterosexual) mentioned taking antidepressants and being stressed due to being abroad and away from family. These accounts show how the pandemic impacted mental health and reduced sexual desire. Antidepressant use, isolation, and uncertainty limited participants’ ability to seek or enjoy pleasure, revealing the link between mental health and sexual well-being.

In addition to emotional distress, physical illness also played a role. Some participants said that their sex drive had decreased after having experienced SARS-CoV-2 infection. A few participants, like Participant 1 (25, bicurious), reported “no desire for sex” and “zero sex drive.” The impact of the COVID-19 pandemic and subsequent lockdowns on participants’ masturbation frequency varied depending on factors such as living situation and access to privacy. For some, masturbation frequency increased when India’s first lockdown was announced. As the pandemic worsened and restrictions tightened, participants’ masturbation frequency decreased. Some participants’ masturbation frequency fluctuated depending on living conditions and privacy. Participant 12 (33, heterosexual) said, “pressure at work decreased my frequency.” Participant 8 (25, heterosexual) said their frequency rose during the first, relaxed lockdown, but decreased during the second, more stressful wave. Participant 5 (29, bicurious) said her masturbation frequency changed with lockdown privacy levels, “When I lived alone, I masturbated more. Now I have more freedom, like wearing whatever I want at home.” Two participants reported no change in their sexual life during lockdown, as both were single. These variations suggest that sexual desire is shaped not just by emotional states but also by external structural conditions like living arrangements, work stress, and health status.

Overall, this subtheme reflects how sexual desire is a fluctuating experience, shaped by emotional distress, physical and mental health, and environmental conditions. Participants’ accounts highlight how crises shift priorities and reveal the interplay of mental health, structural stability, and sexual desire.

##### Subtheme 3: Re-inventing Ways for Pleasure

This sub-theme captures participants’ new ways of exploring pleasure despite pandemic restrictions. Some reported trying phone sex, sexting, and online dating, while others shifted toward masturbation. This sub-theme reflects how the pandemic enabled participants to experiment with solitude, using isolation as an opportunity to reclaim agency and redefine pleasure.

A few participants decided to explore masturbation for the first time during the pandemic. They shared that they had been alone and did not have a partner for sexual activity. Hence, they felt like they had to resort to self-pleasure and masturbation to satisfy their sexual urges. Participant 10 (25, heterosexual) mentioned, “So initially I was like so frustrated because I always had someone who did things for me,” but eventually it got easy, and she described feeling comfortable with masturbation. Similarly, Participant 18 (33, heterosexual), a doctor, described being too busy to meet someone for dating and saw this period as a time for “self-discovery and self-satisfaction.” Others, like Participant 14 (30, heterosexual), described trying online dating (Bumble) for the first time during the pandemic. Participant 17 (30, heterosexual and bicurious) described exploring phone or video sex with her partner through WhatsApp and mentioned “Doing sex after a long time, over the phone, was much more fun. Doing it every day gives a zing to the whole relationship.” Overall, this sub-theme reveals how participants adapted to the constraints of the pandemic by turning inward and embracing solitary or virtual forms of pleasure. These sexual acts served as ways to reclaim agency, reframe pleasure, and meet their needs on their own terms. Overall, this sub-theme shows how participants adapted to pandemic constraints by turning inwards to reclaim agency, redefine pleasure, and meet their own needs.

## Discussion

### Summary of Findings

Using qualitative interviews, we examined Indian women’s experiences of masturbation during the COVID-19 pandemic, focusing on how they learned about masturbation, their emotional responses, and how pandemic-related restrictions shaped their sexual experiences. Participants reported limited access to sexual knowledge, often relying on informal sources such as the internet, friends, or partners due to the absence of formal, pleasure-affirming sex education. We found that this gap in sexual knowledge, shaped by class, geography, and privacy, contributed to participants’ guilt and fear around masturbation. Participants reported that early messaging from social institutions, including school, family, religion, and society, framed women’s sexual pleasure as dangerous or immoral, especially for unmarried women. We found that this messaging reinforced participants’ shame and guilt, leading to limiting self-exploration. Over time, some participants reported moving from shame to acceptance. We found that the COVID-19 pandemic exacerbated these structural barriers by reducing privacy, leading to reduced partnered sex, increasing emotional distress, and family monitoring. However, some participants navigated these constraints by exploring pleasure through masturbation and digital sex. Our findings reveal how intersecting cultural, familial, and pandemic-related factors shaped participants’ experiences of masturbation, with some resisting constraints to reclaim pleasure through self-exploration.

### Key Contributions

Existing research has established that Indian women face limited access to sexuality education, sociocultural regulation of sexual behavior, and stigma surrounding women’s desire (Abraham, 2001; Kar & Koola, 2007; Sachdev, 1998; Sharma & Sharma, 1998). The previous literature has largely centered partnered or marital sexuality and relied on quantitative surveys except a recent study (Singh et al., 2025). As a result, little is known about how Indian women learn about masturbation, how they emotionally navigate shame and pleasure over time, or how structural disruption, such as the COVID-19 pandemic shaped masturbation practices. Our study addresses these gaps by centering masturbation among Indian women, which is a topic largely absent from Indian sexual and reproductive health discourse, which continues to prioritize risk, reproduction, and partnered sex over individual self-pleasure (Edmunds & Gupta, 2016; Sawant et al., 2024; Unnithan, 2022). By focusing on masturbation, we challenge dominant frameworks that restrict women’s sexuality to procreation and instead emphasize that self-pleasure as an individual activity is an essential part of sexual well-being. Our findings extend prior research by showing that reliance on informal sex education shapes not only misinformation, but also emotions of guilt, fear, and delayed self-acceptance around masturbation. We build on previous research showing how fear-based messaging from schools, religious institutions, and families shape early guilt around women’s pleasure. Our findings show a gradual emotional shift from shame toward acceptance. Our findings show that women’s masturbation is shaped by the joint effects of education gaps, family surveillance, cultural shame, and privacy constraints, rather than isolated influences. Finally, our findings demonstrate that the COVID-19 pandemic functioned not only as a constraint on women’s sexual lives, but also as a context in which some women reclaimed pleasure through solitary and digital practices.

First, our findings extend prior research that the lack of pleasure-affirming sex education not only forces women to rely on informal sources that often misrepresent sexual pleasure, but also leading to confusion, guilt, and shame around masturbation and sexual development. Although sexual pleasure is a key motivator for sexual activity (Ford et al., 2019), most participants reported receiving little to no sexuality education at home or school. This experience reflects the national trends, where over 71% of Indian youth report not receiving sexuality education from parents or teachers (Pandey & Rao, 2023). Many Indian states have actively banned school-based sex education, citing concerns that it promotes early sexual behavior and undermines traditional values (Ismail et al., 2015; Pandey & Rao, 2023). We found that many participants primarily learned about masturbation through informal sources such as the internet and peers, consistent with prior research with young Indians aged 17–22 (Joshi, 2010; Shekar et al., 2023; Sur, 2021). These informal sources often reinforced misinformation and unrealistic depictions of women’s pleasure (Chertow, 2019; Edmunds & Gupta, 2016). Many participants described experiencing sexual pleasure before they had the language to understand it, highlighting a disconnect between bodily experience and available sexual knowledge. The gap in sex education leaves women isolated and misinformed, producing shame and limiting sexual agency. Second, our findings reinforce and broaden existing knowledge by showing how cultural norms and family surveillance operate simultaneously to stigmatize women’s desire and restrict self-exploration, reinforcing shame, confusion, and self-doubt. Aligned with previous literature, we found that cultural norms expect unmarried women to remain abstinent, linking their virginity to family honor (Abraham, 2001; Chakraborty & Thakurata, 2013; Hunjan & Towson, 2007; Subaiya, 2024). These expectations, enforced through family and societal surveillance, stigmatize women’s desire, restrict sexual exploration, and prioritize male-centered models of pleasure (Dasgupta, 2003; Heydari et al., 2021; Pereira et al., 2007). One participant shared, “I thought something was wrong with me as this [masturbation] was the only way I could get pleasure. I don’t get pleasure out of penetration.” This reflection highlights how dominant sexual scripts that center penetrative heterosexual sex can marginalize women’s diverse desires. In addition to these cultural and familial constraints, Indian women also face structural barriers, as the legal and policy environment implicitly criminalizes sexual tools like sex toys, limiting access to pleasure and reinforcing the notion that women’s masturbation is unlawful (Mhatre et al., 2025). Participants’ experiences demonstrate how overlapping systems of cultural, familial, and structural oppression silence women’s experiences of masturbation and sexual pleasure, reinforcing confusion, shame, and self-doubt.

Third, our findings build on prior research by showing that participants initially experienced shame around masturbation due to fear-based messaging, but over time, many developed self-acceptance through personal growth and changing contexts. Consistent with prior studies, our findings show that early abstinent and fear-based messaging often framed masturbation as immoral or sinful, which led to early feelings of shame and guilt among participants (Hunt & Jung, 2009; Mahajan et al., 2013; Rajan & Pathak, 2024). Consistent with prior studies, we found that family responses reinforced shame by enacting sexual surveillance from an early age (Dodia, 2023; Edmunds & Gupta, 2016; Rajan & Pathak, 2024; Sur, 2021; Viswanathan, 2024). Our study’s unique finding is that many participants described a gradual shift from shame and guilt toward acceptance and self-compassion as they aged. Our findings show that changing social contexts and personal growth can reshape women’s emotional relationships with their sexuality.

Lastly, our findings deepen existing analysis of COVID-19’s impact on sexual activity by showing that the pandemic both reinforced social constraints and opened opportunities for self-exploration. Several participants reported reduced masturbation and partnered sex due to lack of privacy, familial surveillance, and emotional distress, aligning with global multi-country reviews on decline in sexual activity during the pandemic (Masoudi et al., 2022; Qaderi et al., 2023). However, few used this period of isolation as an opportunity to experiment, such as phone sex and self-pleasure. These findings reflect broader studies on the rise of digital sexual behaviors and how solitude enabled new forms of sexual engagement (Banerjee & Rao, 2021; Döring, 2020; Qalati et al., 2022). Consistent with Mhatre et al., (2025) some participants used this disruption and isolation as a chance to explore autonomy and desire, highlighting that even in crisis, women can reframe constraints as moments for agency (Sur, 2021).

Overall, our findings show that while existing literature highlights the role of inadequate sex education, sociocultural norms, and fear-based messaging in shaping participants’ guilt and shame around masturbation, we add that these forces jointly restrict self-exploration, and that some participants’ emotions shifted from guilt to self-acceptance over time, with the pandemic both deepening social constraints and opening new methods of sexual communication for pleasure.

### Strengths

By studying women’s masturbation experiences, we centered the neglected narratives of Indian women’s experiences of sexual pleasure, a topic marginalized in both public discourse and sexuality research. Conducting the study during the COVID-19 pandemic provides a unique setting for understanding the participants disrupted sexual lives. Previous research on Indian women’s sexual health has primarily relied on surveys, medicalized frameworks, and focused on sexual pleasure in a relational or marital context. In contrast, we centered unmarried women’s voices through in-depth interviews, allowing for a richer understanding of their emotions and lived experiences. The team’s lived experience within Indian cultural contexts enhanced our ability to interpret and contextualize the findings. Rigor was strengthened by collaborative coding with multiple coders (Braun & Clarke, 2022). We captured diverse voices by including participants across regions, sexual orientations, and relationship statuses. Conducting interviews in English, Hindi, and Marathi helped ensure the inclusion of participants from different linguistic and cultural backgrounds, broadening the study’s reach and relevance. Overall, by centering unmarried women’s narratives across diverse contexts and using culturally informed and linguistically inclusive methods, this study advances a sociocultural understanding of women’s sexual pleasure in India.

### Limitations

This study’s limitations underscore the challenges of capturing the diversity of Indian women’s sexual experiences. Our sample was relatively small, composed mainly of young, cisgender, heterosexual, English-speaking, highly educated, able-bodied, and middle-class women. Our sample did not include trans, non-binary, or gender-diverse individuals, and thus our findings may not capture the full spectrum of masturbation experiences among Indian women. Most participants were from urban or semi-urban settings, limiting the study’s ability to capture the geographical diversity of sexual norms, privacy constraints, and access to sexual health resources across India. The use of snowball sampling and online recruitment likely contributed to this homogeneity, potentially excluding women with more conservative views or limited digital access. Conducting interviews via Zoom during the COVID-19 pandemic may have excluded participants with restricted internet access or phone ownership, especially younger or unmarried women in rural areas. Although the sample included women who identified as bi-curious and pansexual, most participants discussed sexual experiences within heterosexual relationships, likely reflecting underreporting rather than the absence of non-heterosexual experiences given stigma and limited safe spaces for queer women in India. As the interviewer was an Indian cisgender woman, participants may have felt hesitant to disclose non-heterosexual experiences, which are considered taboo in India. In addition to the interviewer’s positionality, the interview guide’s focus on masturbation rather than partnered relationships further limited disclosure. Limited data meant we could not compare experiences across sexual identities and should not treat women’s experiences as the same. Although we did not include prior masturbation as an inclusion criterion for this larger study on the sexual lives of Indian women, all participants reported having masturbated at least once. This suggests possible self-selection, as women who felt more comfortable with sexual exploration or/and discussing it with a researcher may have been more likely to participate. As a result, the study does not capture the thoughts, beliefs, or curiosities about masturbation among Indian women who have never masturbated. Future research should examine these perspectives. Finally, the study’s cross-sectional design limited our ability to explore changes in sexual experiences over time. Future studies should use more inclusive sampling strategies, consider repeat interviews for greater depth, and center the experiences of queer, rural, disabled, and older women to reflect better the diversity of Indian women’s sexual lives (Goyes & Sandberg, 2024). These limitations highlight the need for future research to adopt intersectional, inclusive, and longitudinal approaches.

### Implications

Our findings have implications for public health, sexuality education, and future research on women’s sexual pleasure in India and South Asia. Globally, there is growing recognition that sexual pleasure is a critical component of sexual and reproductive health (Starrs et al., 2018). Yet, public health frameworks exclude women’s sexual pleasure, reinforcing patriarchal norms that prioritize male sexual needs, whereas centering pleasure can help challenge cultural stigma surrounding women’s sexuality (Kalra & Bhugra, 2013; Klein et al., 2022). When women are aware of their desires and feel entitled to pleasure, they are more likely to engage in safe, consensual, and satisfying sexual relationships (Beaulieu et al., 2023; Fahs & Swank, 2013; Kettrey, 2018). Our findings underscore the urgent need for accessible, accurate, and judgment-free information on masturbation in India, especially in regional Indian languages and across digital and offline platforms. Social media emerged as a common learning source for masturbation and can be used as a platform for normalizing masturbation and countering myths. Fine and McClelland (Fine & Mcclelland, 2006) have called for sexuality education frameworks that go beyond risk-based approaches and emphasize the positive dimensions of sexuality, making discussions of pleasure part of the normative discourse. Initiatives by Indian organizations such as Agents of Ishq (Vohra, n.d.) and Love Matters (Yadav, 2011) have laid the groundwork by offering body-positive, queer-affirming, and pleasure-oriented content. However, their reach remains limited to digitally connected youth, excluding middle-aged and older women or those in smaller or rural towns with restricted phone access (GSMA, 2024). To advance sexual health equity, public health initiatives may include pleasure-affirming content in culturally grounded sexuality education. This approach can challenge harmful myths, reduce shame, and affirm women’s right to sexual pleasure.

While mainstream diagnostic models often neglect the sociocultural context of women’s sexuality in India, our findings emphasize the need for culturally sensitive tools and therapeutic approaches that center sexual pleasure as vital to women’s well-being (Sharma & Khanna, 2021). Participants identified online sources as their primary source of sexual knowledge. Mainstream pornography often reinforces the expectations that “sex is only penetration” or “only vaginal sex leads to orgasm” (Gavey et al., 1999). Expanding women’s access to feminist and ethical porn may help broaden diverse understandings of sexual pleasure.

Future research may adopt intersectional approaches and expand sample diversity across age, caste, class, region, disability, religion, and sexual orientation. A more inclusive understanding of sexual pleasure would include queer-centered research that moves beyond heteronormative frameworks and documents how queer women uniquely experience and make meaning of sexuality. Studies involving couples could help understand how mutual and individual pleasure are negotiated in relationships. Expanding this study to include other South Asian countries like Pakistan and Bangladesh would enable regional comparisons and deeper insight into how similar sociocultural and patriarchal structures shape women’s sexual pleasure and agency. Finally, large-scale surveys using standardized sexual pleasure measures would support comparative, policy-relevant insights into women’s sexual well-being. Centering pleasure within culturally relevant frameworks, expanding access to inclusive sex education, and diversifying future research are critical steps toward promoting sexual well-being for all women.

### Conclusion

In our goal to examine the masturbation experiences of Indian women during the COVID-19 pandemic, we found that our participants’ experiences of masturbation were shaped by uneven access to sexual knowledge, cultural silence around women’s sexuality, and societal and familial surveillance. Despite these social constraints, participants found ways to reclaim sexual agency and pleasure, especially during the COVID-19 pandemic. By centering women’s narratives on masturbation, our findings challenge the dominant sexual health discourses that frame women’s sexuality around risk, reproduction, and marriage. We also challenge the Indian cultural silence and stigma around women’s masturbation by showing that it is not just a private act, but a sociocultural practice through which participants asserted sexual agency. Our findings highlight the need for pleasure-affirming sex education in schools, for clinicians to nurture nonjudgmental spaces and diagnostic tools that adapt to sociocultural context, and for policymakers to promote women’s sexual pleasure and masturbation as a critical part of health frameworks. Building on our findings, future research may include diverse identities to capture the complexity of sexual pleasure across India.

## Data Availability

Given the confidential nature of the qualitative interview data, the raw qualitative data are not available outside of the research team.

## Appendix

### Semi-Structured Interview Guide

It’s nice to meet you. My name is _________ and I am a (Doctoral student) in the ______ (School/Department) at the Indiana University, USA.

I’m part of a larger research team that studies human sexuality including the many different ways that people experience their sexual desires, experiences, and behaviors. We appreciate you scheduling time to learn more about the study. You may remember that we sent you a copy of the Study Information Sheet. Here is a PDF copy of the Study Information Sheet. It describes that participation in these interviews is completely voluntary and that the interviews are expected to take about 60–90 min. If you choose to participate, you can skip any questions you want. Interviews are recorded, transcribed, and then the recordings are deleted. If you choose to participate, then in the transcripts we will change your name to a pretend name. We will also change the names of anyone you reference. You can choose your own pretend name, or we can choose one for you. Participants are given a $25 Amazon international gift card for their participation at the end of the interview.

Please take a few moments to read through the Study Information Sheet and ask me any questions you may have. If you choose to participate, let me know and we can begin the Interview.

Language preference.

[Interviewer pauses while the individual reads through the form and asks any questions.]

For those who agree to participate:

## Interview Begins

Okay, I will now turn on the recording. (recorder turned on).

To confirm, do you agree to participate in the research?

## Introduction

1. How old are you?
2. How would you describe your gender? Assigned as a female and identify as female? Or you anything other than this?
3. How would you describe your sexual identity?
4. What do you do career wise a homemaker/student/business owner/professional or something else?
5. Do you have a pretend name you’d like us to use for your interview or should our research team make one up for you?

## Learning about Masturbation

1. How important is sexual life and sexual health for you?
2. Tell me about the time when you became sexually active? Age, with partner, alone, situations, emotions
3. How did the idea and practice of sexual pleasure came into your life? Age to masturbate, self interest or some one told or something you saw or read, journey from that time to now, any changes, why do you think those changes happened?
4. Tell me your earliest memory about masturbation? Place, people, mindset, time, how it ends, how did you feel. Story? Did you have privacy?
5. Any people who taught or told you about masturbation?
6. Have you ever lived in any country apart from India? If yes, where and for how long?
7. Did that experience change your behavior or perception toward masturbation and sexual pleasure?
8. Any changes happened in perception toward masturbation or sexual pleasure by migrating within India?
9. How much internet has influenced in this journey?

## Individual Masturbation Characteristics

1. Tell me about how do you usually masturbate? What comes to your mind when u hear word masturbate and sexual pleasure.
2. Memory of an excellent and/or worst scenario when you think about masturbation?
3. Do you have any preferred time and place to engage in masturbation? Alcohol?
4. What triggers you to masturbate usually?
5. How many time do you orgasm?
6. Do you use any physical objects (electronic/ non electronic i.e., vibrator, dildo) during masturbation?
7. Do you watch/ hear/ read pornographic material/ erotica during or prior to masturbation?
8. What body parts are involved during masturbation? Vagina, breasts, anus, finger, mouth etc.
9. Where do you feel most pleasurable? clitoris, labial folds, vaginal walls, anal opening, breast, nipples or any other?
10. How long does masturbation last on average?
11. Would you like to share your stories on Orgasm stories? Or your sexual journey? How often do you reach orgasm.

## Masturbating with Partner

1. How frequently do you masturbate alone or/and with partner? (times per month or week)
2. Do you talk about it with your partner? How does usually go? Accepting or judgmental.
3. Memory of one good or bad conversation with your partner?
4. In person masturbation with partner or online?

## Masturbation and COVID-19

1. Grief and loss of people how that affected their desire?
2. Were you suffering yourself? Were you alone?
3. Feeling wise changed in sexual behavior?
4. Economic struggle has changed any behavior?
5. How has frequency of masturbation changed since the COVID-19 pandemic related lockdown?
6. How has your privacy changed since COVID-19?
7. Are there any changes in your sexual practices such as Masturbation, sex, plays since COVID-19? If yes, then what kind of changes?

Those are all the questions we have.

## Conclusion

Is there anything we didn’t ask but should have?

Is there anything else you’d like to add or make sure that we know?

What kinds of questions do you have for me?

Thank you again for your time. Sending you gift card link in via your preferred method of communication. As we discussed, we will have the recording transcribed. We will change your name in the transcript and then delete the recording. Thank you again for participating in our research.

## Code Availability

N/A.
